# Improved simulation of cryogenic fluid mixing at supercritical pressures

**DOI:** 10.1371/journal.pone.0277711

**Published:** 2023-01-19

**Authors:** Muhammad Omair, Hasan U. Akay

**Affiliations:** 1 Centre for Excellence in Science and Technology (CESAT), Islamabad, Pakistan; 2 Graduate School of Natural and Applied Sciences, Atilim University, Ankara, Turkey; Tongji University, CHINA

## Abstract

The combustion chamber pressure of rockets, gas turbines and diesel engines is known to be above the critical pressure of fuel and oxidizers. In the case of rocket engines the fuel and/or oxidizer is often injected at cryogenic temperatures. This elevated combustion chamber pressure and low temperature demands special treatment for numerical analysis of mixing. Thus a novel implementation of an improved equation of state has been proposed which provides better estimation of densities. Experimental and numerical data from literature has been used for validation of the analysis methodology.

## Introduction

**Fig 1:**
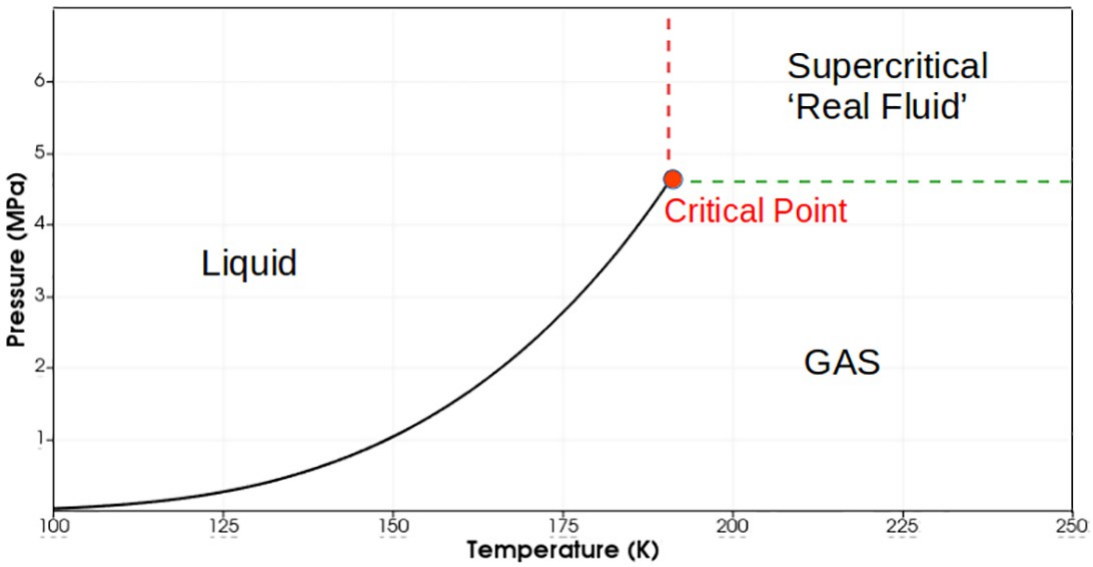
Saturation line of Methane showing the supercritical regime. (Critical temperature and pressure of Methane: 190.564 K, 4.599 MPa) [National Institute of Standards and Technology (NIST) Data] [5].

**Fig 2:**
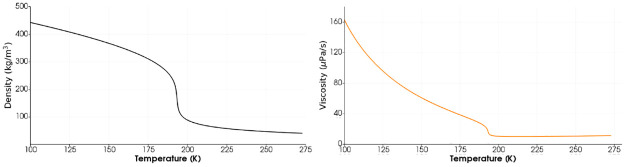
Nonlinear properties of Real fluids. The nonlinear trends in density and viscosity of Methane versus temperature at supercritical pressure of 5 MPa [National Institute of Standards and Technology (NIST) Data] [5].

The modeling of fluid injection and mixing in the diesel engines, gas turbines and rocket engines poses various challenges. The combustion chamber pressure in these systems often exceeds the critical pressure of fuel and oxidizer. The combustion chamber pressures of rocket engines in particular are supercritical for propellants which are commonly injected at cryogenic temperatures. The understanding of injection and mixing of fuel and oxidizer in these complex environments is significant for improving the performance of these systems. Fig 1 illustrates the supercritical region of fluids. Experimental works by Mayer et al. [1], Oschwald & Schik [2], Chehroudi et al. [3] and Habiballah et al. [4] showed that the injected propellant under supercritical pressures behaves like a single-phase fluid without exhibiting atomization and evaporation. Fluid at such pressures has the mixed properties of gases and liquids and shows complex dense gas/gas mixing which is highly sensitive to the pressure and temperature gradients. This results from the repulsive atomic forces which become significant due to high compression. The thermophysical properties of this “Real fluid” are highly nonlinear as shown in Fig 2. The real fluid models employed for such complex environments lack in accurate density estimation as reported by [6]. In this study we have implemented an improved real fluid model using open-source software OpenFOAM to resolve this problem.

## Methodology

### Real fluid model

The simulation of supercritical flows poses a variety of challenges. Fluid behaviour under such conditions is dominated by intermolecular forces. Therefore a specialized equation of state is needed to model the pressure-temperature-density relationship. Van der Waal’s work in developing the quadratic equation of state provided the early basis for including such effects. Peng and Robinson modified van der Waal’s equation to develop a cubic equation of state in 1974 with improved density prediction for fluids especially in liquid [7]. Peng-Robinson Equation of State (PREOS) is given as:

$$\begin{array}{c} P=\frac{RT}{V_{m}-b}-\frac{a\alpha }{V_{m}^{2}+2bV_{m}-b^{2}} \end{array}$$ (1)

$$\begin{array}{c} a\approx 0.45724\frac{R^{2}T_{c}^{2}}{P_{c}} \end{array}$$

$$\begin{array}{c} b\approx 0.0778\frac{RT_{c}}{P_{c}} \end{array}$$

$$\begin{array}{c} \alpha =(1+k\left(1-\sqrt{T_{r}}\right))2 \end{array}$$

$$\begin{array}{c} T_{r}=\frac{T}{T_{c}} \end{array}$$

$$\begin{array}{c} k\approx 0.37464+1.54226\;\omega -0.26992\;\omega^{2} \end{array}$$

Whereas in Eq 1:

*P*: Absolute pressure

*T*: Absolute temperature

*P_c_*: Critical pressure

*T_c_*: Critical temperature

*T_r_*: Reduced temperature

*R*: Universal gas constant (= 8.3145 J/mol K)

*V_m_*: Molar Volume

*ω*: Acentric factor, measure of non-sphericity of molecules

*α*: Soave’s function

PREOS has been extensively used by researchers to model fluids under transcritical and supercritical conditions (Müller [8], Cutrone [9, 10], Hickey [11]). However the density estimation using the classical PREOS is not highly accurate especially for fluids at high pressures and low temperatures. There have been several attempts to improve the performance of PREOS in the past. Forero and Velasquez developed an improved version of Peng-Robinson equation of state (FVPREOS) for accurate estimation of density [12] of non-polar and polar substances at a wide range of pressures. We have utilized this equation of state in the present work. This modified version of Peng-Robinson EOS gives better estimation of densities at a wide range of pressures. Soave alpha function in original PREOS has been replaced with Heyen’s alpha function in FVPREOS:

$$\begin{array}{c} \alpha \left(T\right)=exp[m_{H}\left(1-T_{r}^{n_{H}}\right)] \end{array}$$ (2)

*m_H_* and *n_H_* are determined by minimizing the average absolute relative deviation in vapor pressure.

Forero and Velasquez improved the liquid density estimation by translating the modified equation of state in molar volume.

$$\begin{array}{c} V_{mt}=V_{m}-c \end{array}$$ (3)

Where V_mt_ is the translated molar volume.

For gases and hydrocarbons, the translated volume c has been generalized as:

$$\begin{array}{c} c=\left(0.03209\;\omega -0.01160\right)\frac{RT_{c}}{P_{c}} \end{array}$$ (4)

**Fig 3:**
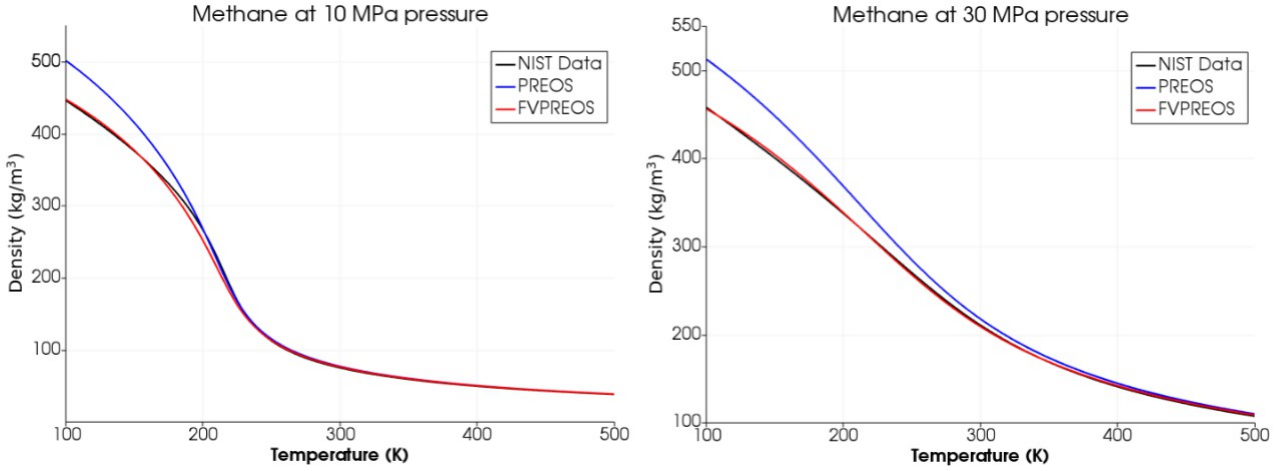
Methane density estimation using FVPREOS. Comparison with experimental data from NIST and PREOS based estimations [5].

**Fig 4:**
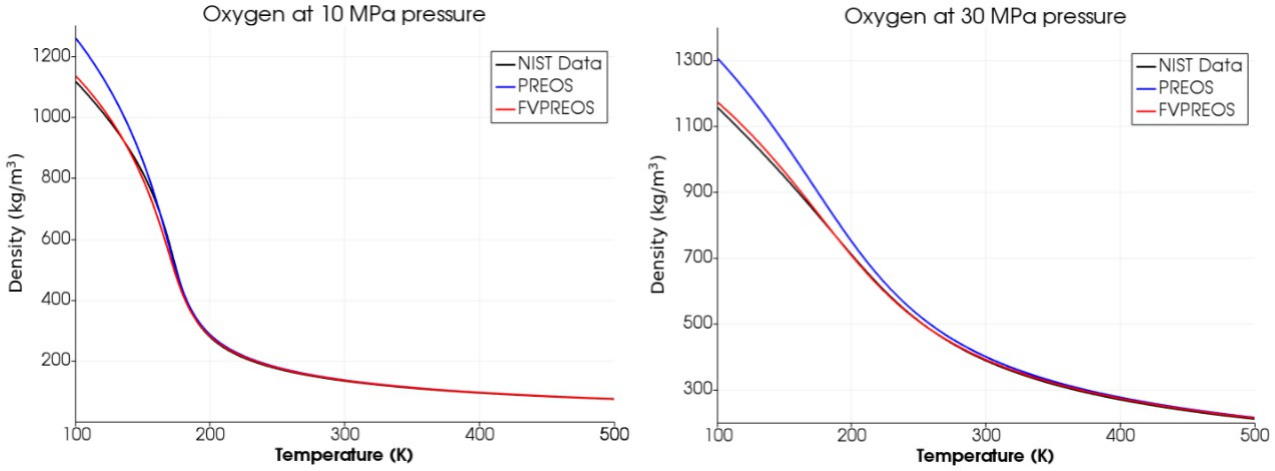
Oxygen density estimation using FVPREOS. Comparison with experimental data from NIST and PREOS based estimations [5].

**Fig 5:**
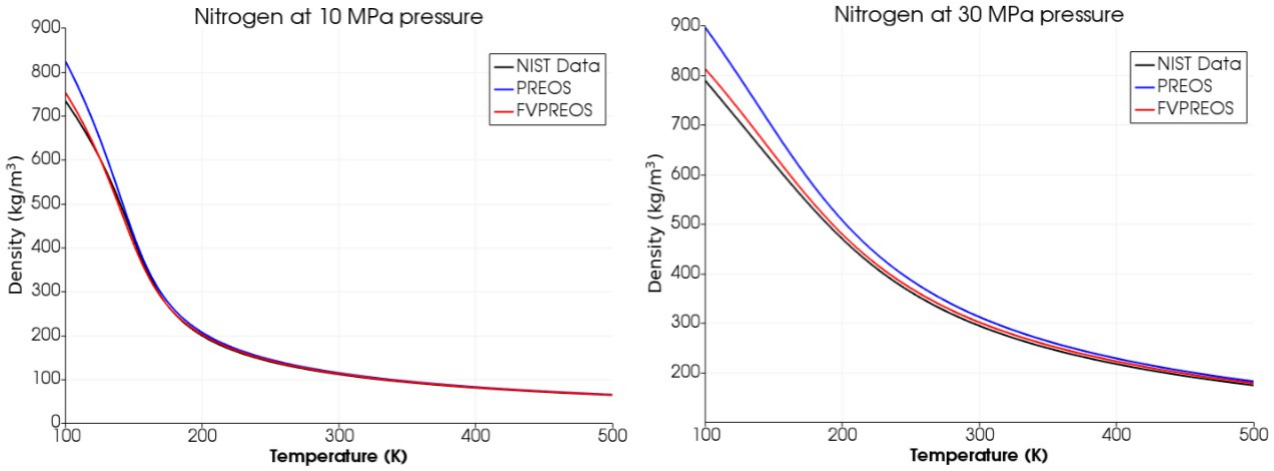
Nitrogen density estimation using FVPREOS. Comparison with experimental data from NIST and PREOS based estimations [5].

We have calculated densities of Methane, Oxygen and Nitrogen using Peng-Robinson equation of state by Forero and Velasquez (FVPREOS) at various pressures. Results have been compared with experimental data from National Institute of Standards and Technology (NIST) and also with the densities calculated using the original Peng-Robinson equation of state (PREOS) as shown in Figs 3–5.

This comparison clearly shows that FVPREOS outperforms original Peng-Robinson equation of state (PREOS) in providing accurate densities under various conditions of pressures and temperature. The pressures of 10 MPa and 30 MPa in above plots are greater than critical pressure for both Oxygen (5 MPa) and Methane (4.6 MPa). FVPREOS performance at higher pressures and lower temperatures makes it eligible for use in simulations of cryogenic propellants mixing at supercritical pressures. This improved equation of state has been implemented in the present work.

### CFD analysis technique

The CFD analysis has been performed with open-source C++ toolbox OpenFOAM. We have added real fluid flow solution capability to “rhoSimpleFoam” solver of OpenFOAM by implementing FVPREOS in the code. The thermophysical models in OpenFOAM were modified for this purpose. The pressure based compressible flow solver employs the SIMPLE (Semi-Implicit Method for Pressure Linked Equations) algorithm. The momentum equation simplified for stationary mesh case is given below:

$$\begin{array}{c} \nabla \cdot (\rho U⊗U)-\nabla \cdot \sigma =S-\nabla P \end{array}$$ (5)

Whereas in Eq 5:

*ρ*: Density

***U***: Velocity

**σ**: Stress tensor

***S***: Source term for momentum

The thermodynamic properties are updated after solving the following energy equation:

$$\begin{array}{c} \nabla \cdot (UE+UP)-\nabla \cdot \dot{q}-\nabla \cdot (U\sigma )=Q \end{array}$$ (6)

Whereas in Eq 6:

*E*: Energy

$\dot{q}$: Diffusive flux of energy

*Q*: Source term for energy

The specie transport parameters of fluids at supercritical pressure exhibit nonlinear behaviour as discussed in the first section. Therefore we have performed polynomial curve fitting for the viscosity and thermal conductivity data obtained from NIST and incorporated the polynomials in our CFD analysis. Then pressure is calculated from the modified continuity equation:

$$\begin{array}{c} \nabla \cdot \left(\rho A_{P}^{-1}\nabla P\right)-\sum_{f}S_{f}\cdot {\left(\rho A_{P}^{-1}H\left(U\right)\right)}_{f}=0 \end{array}$$ (7)

Whereas in Eq 7:

***A***_*P*_ and operator ***H(U)***: Diagonal and off-diagonal components of coefficient matrix resulting from the discretization of momentum equation.

**S**_*f*_: Face area vector.

The reader is referred to [13] for detailed explanation of the terms in this equation. The density is calculated from the pressure by solving the equation of state which is FVPREOS in our case. The turbulence effects on the flow are included by employing the “standard kε-model”.

## Case study

**Fig 6:**
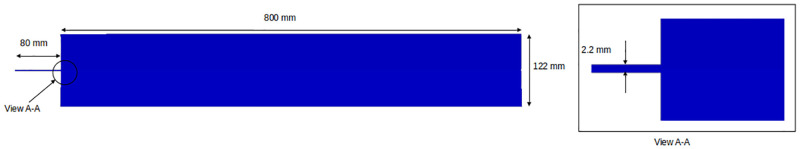
Geometry for analysis.

Experiments from DLR (Deutsches Zentrum für Luft- und Raumfahrt e.V.) Germany have been chosen for validation of our methodology. In these experiments a cryogenic, axisymmetric liquid Nitrogen jet flows into a stagnant environment of gaseous Nitrogen at 298 K through a 2.2 mm diameter (D) injector. Flow densities are measured using Raman scattering method [14, 15]. The geometry of the case is shown in Fig 6.

These experiments have been used for validation of cryogenic fluid study by many research groups. One of the test cases was presented as a test case in the second International Rocket Combustion Modeling workshop, 2001 [16]. This liquid (cryogenic) Nitrogen-gaseous Nitrogen mixing has been used as a sole test case for cryogenic fluid mixing study in the numerical investigations by Cheng et al. [17], Cutrone et al. [9, 10], Schmitt et al. [18], Selle et al. [19], Hickey et al. [11, 20], Niedermeier et al. [21]; Ma et al. [22] and Terashima at al. [23].

We have simulated three test cases from these experiments which are summarized in Table 1.

**Table 1:** Test cases for validation.

Case No.	Chamber Pressure	Injection Jet Velocity	Injection Jet Temperature
1	4 MPa	5.04 m/s	126.9 K
2	3.97 MPa	4.9 m/s	126.9 K
3	3.98 MPa	5.4 m/s	137.0 K

The first test case has been discussed by Hickey and Ihme [11]. They employed an in-house code (CharLES^x^) of Center for Turbulence Research at Stanford University with real fluid model for numerical analysis. Second and third test cases are the third and fourth experimental cases from the experiments of Mayer et al. [15]. In the first and second test cases the injection jet temperature is near the critical point (126.19 K for Nitrogen) whereas in the third case it is higher than the critical temperature. The chamber pressure is higher than the critical pressure (3.398 MPa for Nitrogen) in all cases.

**Fig 7:**
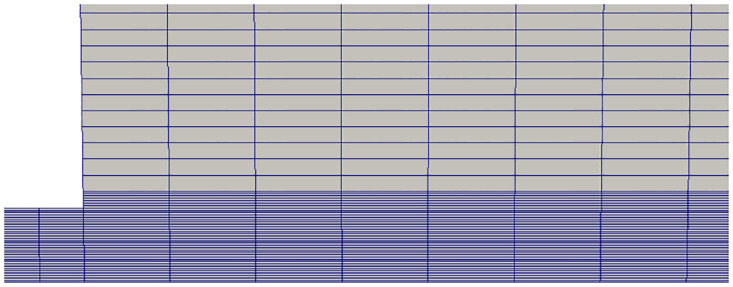
Zoomed view of fine mesh near injector.

**Fig 8:**
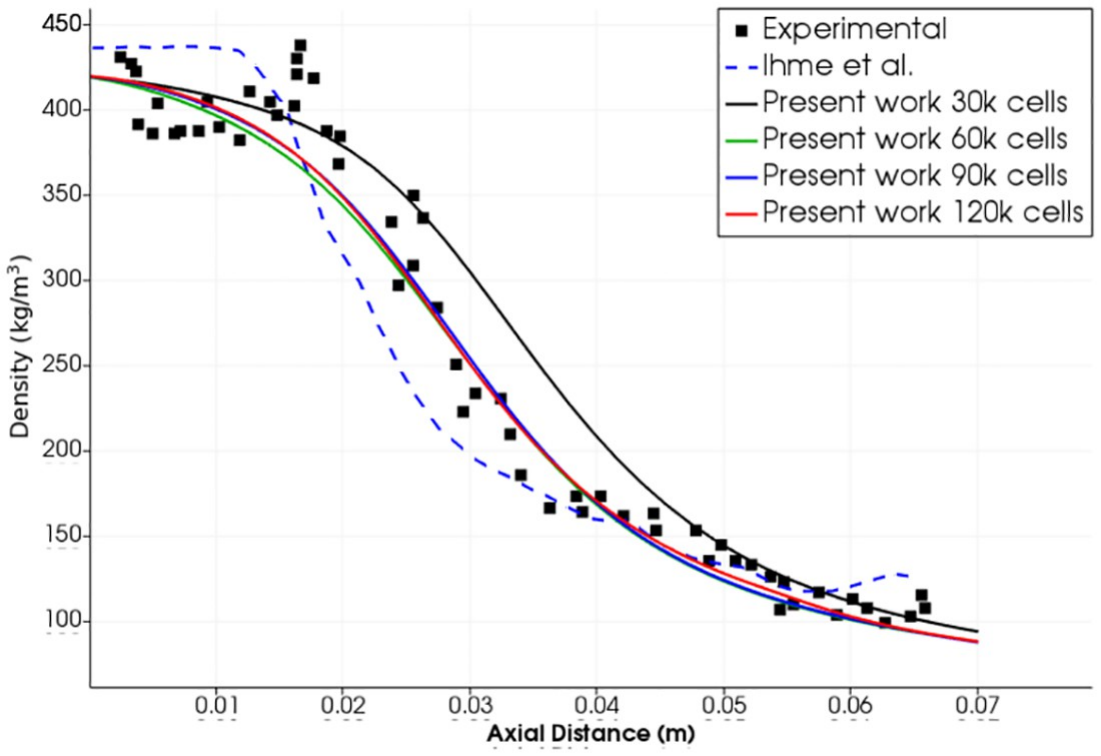
Test case 1. Comparison of density profile along the center-line in present work with reference experimental and numerical results-Injection Temperature: 126.9 K and Injection Velocity: 5.04 m/s [11].

We have solved these cases using our OpenFOAM solver. Axisymmetric geometric model has been used for this analysis as shown in Fig 7. Fig 8 shows our results and comparison with the referenced numerical work for the first test case [11], where the flow densities along the center axis calculated in present work and reference results are presented. Our results conform better with the experimental results as compared with the numerical results of Hickey and Ihme. A grid-convergence study with four different mesh densities suggests that 60,000 cells are sufficient for further work. Maximum difference between numerical and experimental results is in the region with highest density which may be attributed to the optical reflection and refraction of light in this region during Raman scattering testing [17].

The axial density results for test cases 2 and 3 have been compared with the experimental data from Mayer et al. [15] as well as the numerical studies of Selle et al. [19], Ma et al. [22] and Ningegowda et al. [24].

Selle et al. employed the code AVBP of CERFACS (Centre Européen de Recherche et de Formation Avancée en Calcul Scientifique) with PREOS for their work on these test cases [19]. Ma et al. also used the PREOS in their code with double-flux method for trans-critical flows [22], whereas Ningegowda et al. employed the same equation of state in OpenFOAM code for this purpose [24].

**Fig 9:**
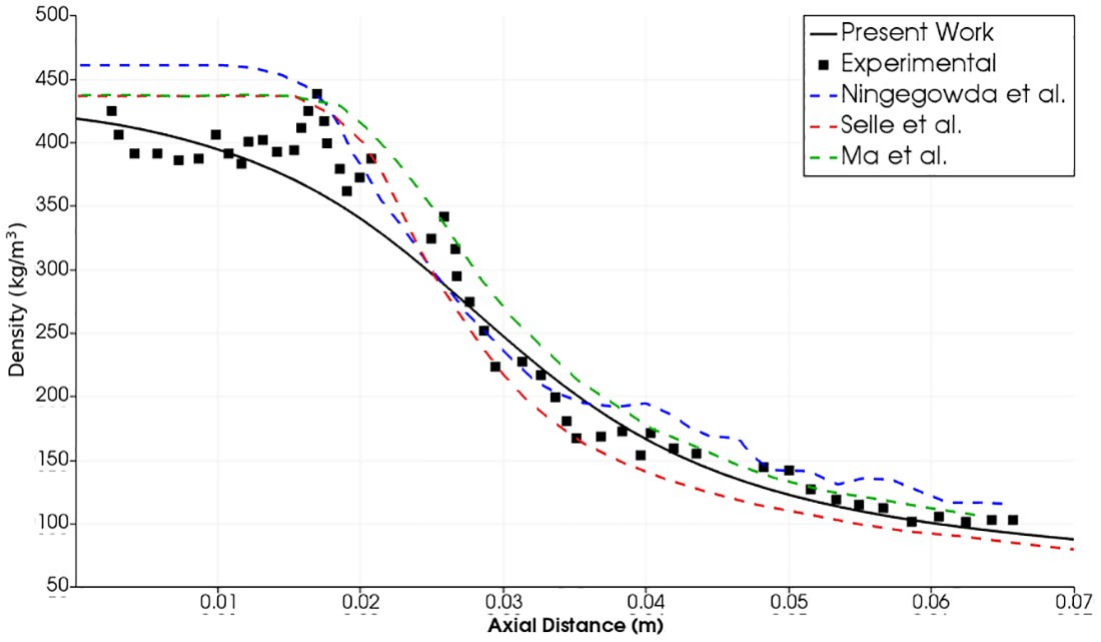
Test case 2. Comparison of density profile along the center-line in present work with reference experimental and numerical results-Injection Temperature: 126.9 K and Injection Velocity: 4.9 m/s [15, 19, 22, 24].

**Fig 10:**
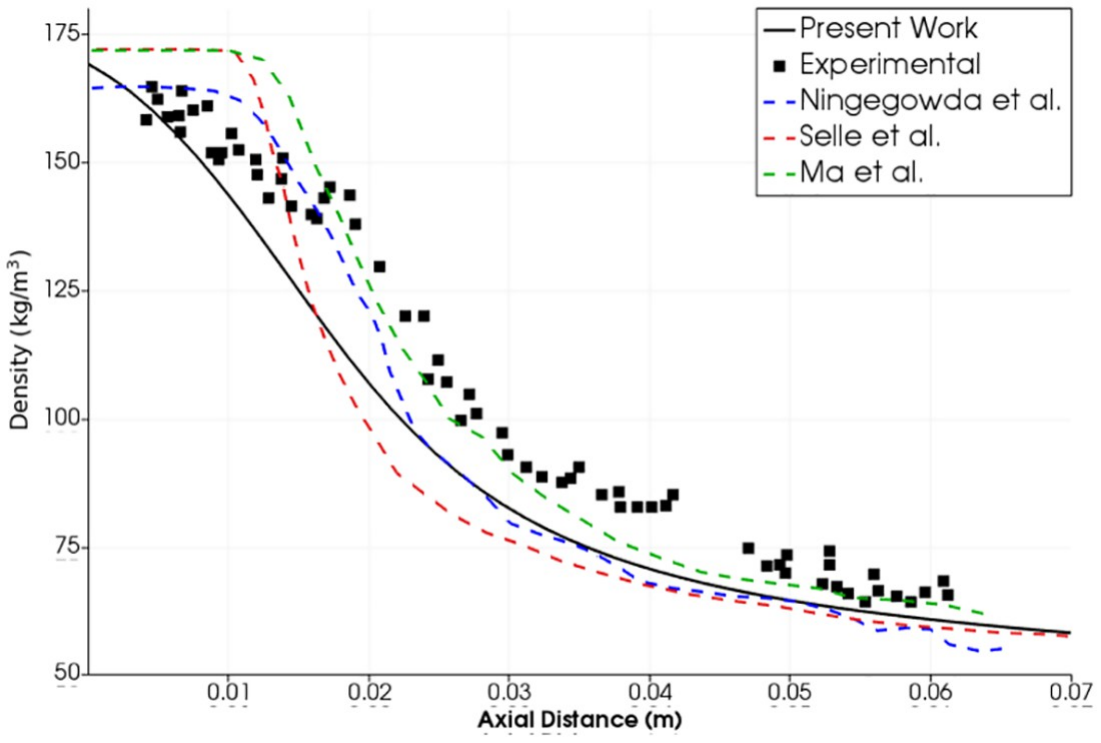
Test case 3. Comparison of density profile along the center-line in present work with reference experimental and numerical results-Injection Temperature: 137 K and Injection Velocity: 5.4 m/s [15, 19, 22, 24].

Reasonable agreements with experimental results have been found in our results with the FVPREOS especially in cases 1 and 2 where the injection temperature is low and near the critical temperature. The results of case 3 also show the validity of our approach, where the early stage of density decay has been captured well. Other numerical studies from literature could not capture this trend. We have simulated the early stage of density decay (0.015*m* > axial distance > 0.005*m*) better than other numerical works in all the three test cases. This is attributed to our implementation of the real fluid model which performs best at cryogenic temperatures in the near-injector zone. In our study the density axial decay at axial distance >0.022*m*(*x*/*D* > 10) accurately follows the experimental one in case 2 (Fig 9). There is difference in our density estimation in the same region in the case 3 compared with the experimental results (Fig 10). However our results are still better than selle et al. [19] and Ningegowda et al. [24] in this region. Moreover the trend of density decay is the same as in the experiment. It is important to note that error bounds on the experimental measurements are not available [11, 17].

## Conclusion

In this study we have developed a new OpenFOAM solver for simulating mixing at cryogenic temperatures in high pressure environments. Our solver is based on real fluid approach as fluids exhibit a single-phase under such conditions. A new equation of state has been employed for this purpose to improve the density estimation in real fluid flows. We have compared our results with results from literature for validation. Our work showcases the worthiness of the new OpenFOAM solver in density estimation at supercritical pressures and cryogenic temperatures. This work will help in better design of combustion chambers especially for rocket engines.

The authors are planning to work further on cryogenics including simulating combustion of such oxidizers and fuels based on the thermodynamic equation of state employed in this manuscript.
